# An Act to Establish a Board of Pharmaceutic Examiners

**Published:** 1881-11

**Authors:** 


### AN ACT

To establish a Board of Pharmaceutic Examiners, and to prescribe the powers and duties of said Board, and to regulate the compounding and vending of medicines, drugs and poisons in the State of Georgia, and to provide a penalty for the infringement of the provisions of this Act.

PREAMBLE.

WHEREAS, in all civilized countries, it has been found necessary to restrict the traffic in medicines and poisons, and to provide by law for the regulation of the delicate and responsible business of compounding and dispensing the powerful agents used in medicines; and whereas, the safety and welfare of the public are endangered by the sale of poisons by unqualified or ignorant persons; and whereas, the power of physicians to overcome disease depends greatly upon their ability to obtain good and unadulterated drugs and skillfully prepared medicines, and the sophistication and adulterations of drugs and medicines is a species of fraud which should be prevented and suitably punished; therefore,

SECTION 1. The General Assembly of the State of Georgia do enact, That, within sixty days after the passage of this Act, the Governor of the State shall appoint five experienced druggists or practical pharmacists, who shall have been actively engaged in the drug business within this State for the last three years immediately preceding their appointment, and these five druggists or practical pharmacists so appointed, shall constitute the Board of Pharmaceutic Examiners, and who shall hold their office for the term of three years, or until their successors shall have been legally appointed and qualified. That three members of said Board at any regular, called or adjourned meeting shall constitute a quorum for the transaction of business. That any vacancy which may occur in said Board by reason of death, resignation or otherwise, shall be filled by the Governor for the unexpired term.

SEC. 2. Be it further enacted, That immediately and before entering upon the duties of said office, the members of said Board shall take the oath prescribed by the constitution of the State for State officers, and shall file the same in the office of the Secretary of State, who, upon receiving the said oaths of office, shall issue to each of said Examiners a certificate of appointment.

SEC. 3. Be it further enacted, That immediately after the appointment and qualification of said Examiners, they shall meet and organize as a Board of Pharmaceutic Examiners, elect a chairman and adopt such rules, regulations and by-laws as they shall deem necessary to carry into execution the provisions of this Act.

SEC. 4. Be it further enacted, That said Board shall meet at least once every twelve months at such place as a majority of the Board may determine, and that the Board may also hold special meetings as frequently and at such places as the proper discharge of the duties shall require; the same to be convened by order of the chairman, and the rules or by-laws shall provide for the giving of proper notice of the time and place of all such meetings to the members of the Board, and to the public.

SEC. 5. Be it further enacted, That it shall be the duty of the said Board to grant licenses: 1st. To pharmacists who, after three years experience in a drug store, kept by a licensed apothecary or pharmacist, have graduated in a college of pharmacy, acknowledged by the American Pharmaceutic Association, and who shall exhibit to the said Board a diploma of the same. 2d. To pharmacists who have obtained a diploma from an authorized foreign college or institution, or examining board, and who shall exhibit the same to the Board of Pharmaceutic Examiners. 3d. To physicians who are graduates of a regular medical college, and who shall exhibit their diplomas to said Board; also to druggists who shall produce to said Board satisfactory evidence of having been engaged in the drug business for a period of ten years next preceding the time of application; also to druggists who have attended a college of pharmacy acknowledged by the American Pharmaceutic Association for at least one term or session, and who have been engaged in the drug business for at least nine (9) years previous to the time of applying for said license. 4th. To druggists who, after three years experience in a drug store kept by a licensed apothecary or pharmacist, shall have passed a satisfactory examination before said Board of Pharmaceutic Examiners. All licenses granted shall be signed by a majority of the whole Board, and shall specify the ground upon which said license is granted, and shall be in such form as the Board shall prescribe.

SEC. 6. Be it further enacted, That all persons applying for examination and license, shall pay to the Board of Pharmaceutic Examiners the sum of fifteen dollars, and if passing the examination shall be furnished with the license as hereinbefore provided, for which no further fee shall be required or paid. Should the applicant fail to stand a satisfactory examination, no fee shall be required for a subsequent examination, such subsequent examination not to be granted within six months after the first. And it shall be the duty of the Board to keep a record of its transactions in a book to be kept for that purpose, by one of its members, said book to be turned over to their successors in office.

SEC. 7. Be it further enacted, That all persons now lawfully engaged in the compounding and vending of medicines, drugs and poisons in this State, shall, on or before December 1st, 1881, and every person who shall be hereafter duly licensed under the provisions of this Act, shall, before engaging in any business under said license, register in the office of the ordinary of the county wherein he resides, or intends to conduct said business, in a book to be kept for that purpose, by said ordinary, his name, nationality and credentials, and date thereof, under which he is entitled to engage in such vocation. For each registration the ordinary shall receive fifty cents, to be paid by the party so registering, and a certificate of such registration, stating the terms of same shall be given him by said ordinary.

SEC. 8. Be it further enacted, That no person shall engage in the compounding or vending of medicines, drugs or poisons, within this State without a full compliance with this Act, except (1) such druggists as are exempt from the operations of the present law by the statutes of the State of Georgia, and such druggists as have heretofore obtained license, and are legally authorized by existing laws, to compound and vend drugs, poisons, and chemicals; (2). Physicians putting up their own prescriptions, and dispensing medicines from their own office; (3). Merchants selling family medicines not poison, as prescribed and allowed by section 1409 of the Code of 1873, of Georgia; (4). Assistants in drug stores where the manager has complied with the requirements of this Act.

SEC 9 Be it further enacted, That any person who shall violate the provisions of either of the two preceding sections of this Act, or shall register fraudulently, shall be guilty of a misdemeanor, and upon conviction shall be punished by fine, not to exceed one hundred dollars, imprisonment, not to exceed three months, either or both, at the discretion of the Board. In all cases of prosecution under this Act, the burden shall be upon the defendant to show his authority.

SEC. 10. Be it further enacted, That all the fees for examination and license, and one-half of the fines collected from convictions under this Act, shall be paid to the Board of Pharmaceutic Examiners, to defray the expenses of the same, and as compensation for their services.

SEC. 11. Be it further enacted, That this Act shall take effect from and after the date of its passage.

SEC. 12. Repeals conflicting laws.

Approved, September 29th, 1881.

